# Racial and ethnic disparities in aortic stenosis within a universal healthcare system characterized by natural language processing for targeted intervention

**DOI:** 10.1093/ehjdh/ztaf018

**Published:** 2025-03-18

**Authors:** Dhruva Biswas, Jack Wu, Sam Brown, Apurva Bharucha, Natalie Fairhurst, George Kaye, Kate Jones, Freya Parker Copeland, Bethan O’Donnell, Daniel Kyle, Tom Searle, Nilesh Pareek, Rafal Dworakowski, Alexandros Papachristidis, Narbeh Melikian, Olaf Wendler, Ranjit Deshpande, Max Baghai, James Galloway, James T Teo, Richard Dobson, Jonathan Byrne, Philip MacCarthy, Ajay M Shah, Mehdi Eskandari, Kevin O’Gallagher

**Affiliations:** School of Cardiovascular and Metabolic Medicine and Sciences, The James Black Centre, King’s College London, 125 Coldharbour Lane, London SE5 9NU, UK; Cardiovascular Department, King’s College Hospital NHS Foundation Trust, Denmark Hill, London SE5 9RS, UK; Section of Cardiovascular Medicine, Department of Internal Medicine, Yale School of Medicine, New Haven, CT, USA; School of Cardiovascular and Metabolic Medicine and Sciences, The James Black Centre, King’s College London, 125 Coldharbour Lane, London SE5 9NU, UK; School of Cardiovascular and Metabolic Medicine and Sciences, The James Black Centre, King’s College London, 125 Coldharbour Lane, London SE5 9NU, UK; Cardiovascular Department, King’s College Hospital NHS Foundation Trust, Denmark Hill, London SE5 9RS, UK; School of Cardiovascular and Metabolic Medicine and Sciences, The James Black Centre, King’s College London, 125 Coldharbour Lane, London SE5 9NU, UK; Cardiovascular Department, King’s College Hospital NHS Foundation Trust, Denmark Hill, London SE5 9RS, UK; Cardiovascular Department, King’s College Hospital NHS Foundation Trust, Denmark Hill, London SE5 9RS, UK; Cardiovascular Department, King’s College Hospital NHS Foundation Trust, Denmark Hill, London SE5 9RS, UK; South London Office of Specialised Services, South London Cardiovascular Network, London, UK; South London Office of Specialised Services, South London Cardiovascular Network, London, UK; South London Office of Specialised Services, South London Cardiovascular Network, London, UK; South London Office of Specialised Services, South London Cardiovascular Network, London, UK; School of Cardiovascular and Metabolic Medicine and Sciences, The James Black Centre, King’s College London, 125 Coldharbour Lane, London SE5 9NU, UK; School of Cardiovascular and Metabolic Medicine and Sciences, The James Black Centre, King’s College London, 125 Coldharbour Lane, London SE5 9NU, UK; Cardiovascular Department, King’s College Hospital NHS Foundation Trust, Denmark Hill, London SE5 9RS, UK; School of Cardiovascular and Metabolic Medicine and Sciences, The James Black Centre, King’s College London, 125 Coldharbour Lane, London SE5 9NU, UK; Cardiovascular Department, King’s College Hospital NHS Foundation Trust, Denmark Hill, London SE5 9RS, UK; School of Cardiovascular and Metabolic Medicine and Sciences, The James Black Centre, King’s College London, 125 Coldharbour Lane, London SE5 9NU, UK; Cardiovascular Department, King’s College Hospital NHS Foundation Trust, Denmark Hill, London SE5 9RS, UK; School of Cardiovascular and Metabolic Medicine and Sciences, The James Black Centre, King’s College London, 125 Coldharbour Lane, London SE5 9NU, UK; Cardiovascular Department, King’s College Hospital NHS Foundation Trust, Denmark Hill, London SE5 9RS, UK; Cardiovascular Department, King’s College Hospital NHS Foundation Trust, Denmark Hill, London SE5 9RS, UK; Department of Cardiovascular Surgery, Cleveland Clinic, London, UK; School of Cardiovascular and Metabolic Medicine and Sciences, The James Black Centre, King’s College London, 125 Coldharbour Lane, London SE5 9NU, UK; Cardiovascular Department, King’s College Hospital NHS Foundation Trust, Denmark Hill, London SE5 9RS, UK; School of Cardiovascular and Metabolic Medicine and Sciences, The James Black Centre, King’s College London, 125 Coldharbour Lane, London SE5 9NU, UK; Cardiovascular Department, King’s College Hospital NHS Foundation Trust, Denmark Hill, London SE5 9RS, UK; Centre for Rheumatic Disease, King’s College London, London, UK; Department of Rheumatology, King’s College Hospital NHS Foundation Trust, London, UK; Institute of Psychiatry, Psychology and Neurosciences, King’s College London, London, UK; Department of Neurology, King’s College Hospital NHS Foundation Trust, London, UK; Institute of Psychiatry, Psychology and Neurosciences, King’s College London, London, UK; School of Cardiovascular and Metabolic Medicine and Sciences, The James Black Centre, King’s College London, 125 Coldharbour Lane, London SE5 9NU, UK; Cardiovascular Department, King’s College Hospital NHS Foundation Trust, Denmark Hill, London SE5 9RS, UK; School of Cardiovascular and Metabolic Medicine and Sciences, The James Black Centre, King’s College London, 125 Coldharbour Lane, London SE5 9NU, UK; Cardiovascular Department, King’s College Hospital NHS Foundation Trust, Denmark Hill, London SE5 9RS, UK; School of Cardiovascular and Metabolic Medicine and Sciences, The James Black Centre, King’s College London, 125 Coldharbour Lane, London SE5 9NU, UK; Cardiovascular Department, King’s College Hospital NHS Foundation Trust, Denmark Hill, London SE5 9RS, UK; School of Cardiovascular and Metabolic Medicine and Sciences, The James Black Centre, King’s College London, 125 Coldharbour Lane, London SE5 9NU, UK; Cardiovascular Department, King’s College Hospital NHS Foundation Trust, Denmark Hill, London SE5 9RS, UK; School of Cardiovascular and Metabolic Medicine and Sciences, The James Black Centre, King’s College London, 125 Coldharbour Lane, London SE5 9NU, UK; Cardiovascular Department, King’s College Hospital NHS Foundation Trust, Denmark Hill, London SE5 9RS, UK

**Keywords:** Aortic stenosis, Artificial intelligence, Transcatheter aortic valve replacement, Ethnicity, Health equity

## Abstract

**Aims:**

Aortic stenosis (AS) is a condition marked by high morbidity and mortality in severe, symptomatic cases without intervention via transcatheter aortic valve implantation (TAVI) or surgical aortic valve replacement (SAVR). Racial and ethnic disparities in access to these treatments have been documented, particularly in North America, where socioeconomic factors such as health insurance confound analyses. This study evaluates disparities in AS management across racial and ethnic groups, accounting for socioeconomic deprivation, using an artificial intelligence (AI) framework.

**Methods and results:**

We conducted a retrospective cohort study using a natural language processing pipeline to analyse both structured and unstructured data from > 1 million patients at a London hospital. Key variables included age, sex, self-reported race and ethnicity, AS severity, and socioeconomic status. The primary outcomes were rates of valvular intervention and all-cause mortality. Among 6967 patients with AS, Black patients were younger, more symptomatic, and more comorbid than White patients. Black patients with objective evidence of AS on echocardiography were less likely to receive a clinical diagnosis than White patients. In severe AS, TAVI and SAVR procedures were performed at lower rates among Black patients than among White patients, with a longer time to SAVR. In multivariate analysis of severe AS, controlling for socioeconomic status, Black patients experienced higher mortality (hazard ratio = 1.42, 95% confidence interval = 1.05–1.92, *P* = 0.02).

**Conclusion:**

An AI framework characterizes racial and ethnic disparities in AS management, which persist in a universal healthcare system, highlighting targets for future healthcare interventions.

## Graphical Abstract

**Graphical Abstract:**
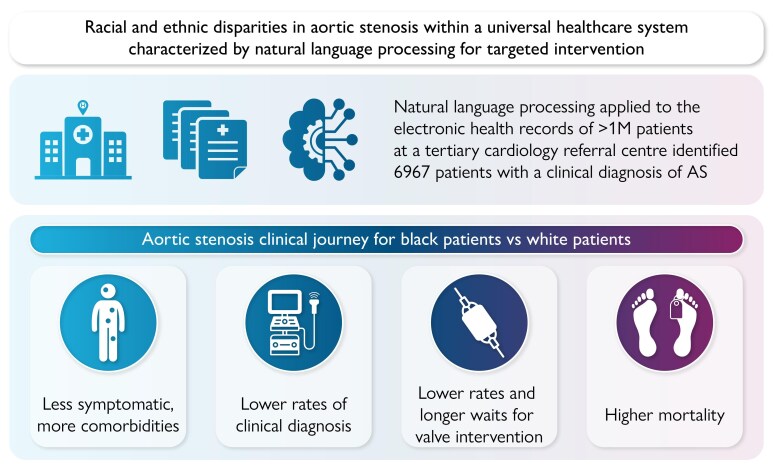

## Introduction

Aortic stenosis (AS) is a common valvulopathy and is associated with considerable morbidity and mortality if untreated.^1,2^ For patients with severe and symptomatic AS, timely diagnosis and valve replacement, either by conventional surgery [surgical aortic valve replacement (SAVR)] or by transcatheter aortic valve implantation (TAVI), is the gold-standard treatment.^3^ Although valve replacement is life-prolonging, these procedures are technologically advanced, costly, and highly regulated, which may give rise to inequities in access. For example, Black patients seem to be under-represented in those undergoing valvular intervention for AS. US studies have described lower SAVR/TAVI rates among Black patients than among White patients,^4,5^ that Black patients were less likely to receive a clinical diagnosis of AS in the presence of positive echocardiogram findings,^6^ and that Black patients were less likely to be referred for TAVI and more likely to be lost to follow-up.^7^ Moreover, recent trial data supporting the use of TAVI in patients with severe AS at low or intermediate surgical risk^8^ may increase eligibility without improving access. Therefore, addressing racial and ethnic disparities in the diagnosis and treatment of AS is an urgent and growing clinical need.

Electronic databases could pinpoint the most effective healthcare interventions for eliminating treatment disparities, paving the way for equitable access to valve replacement.^9^ While existing studies have described disparities either at the point of diagnosis or at treatment, a comprehensive study quantifying racial biases at each stage in the journey of a patient with AS—and linking these to differences in clinical outcome—is lacking. Furthermore, the existing literature is largely drawn from North American cohorts, where it has historically been difficult to adjust for the effects of socioeconomic deprivation and payor biases.

**Figure 1:**
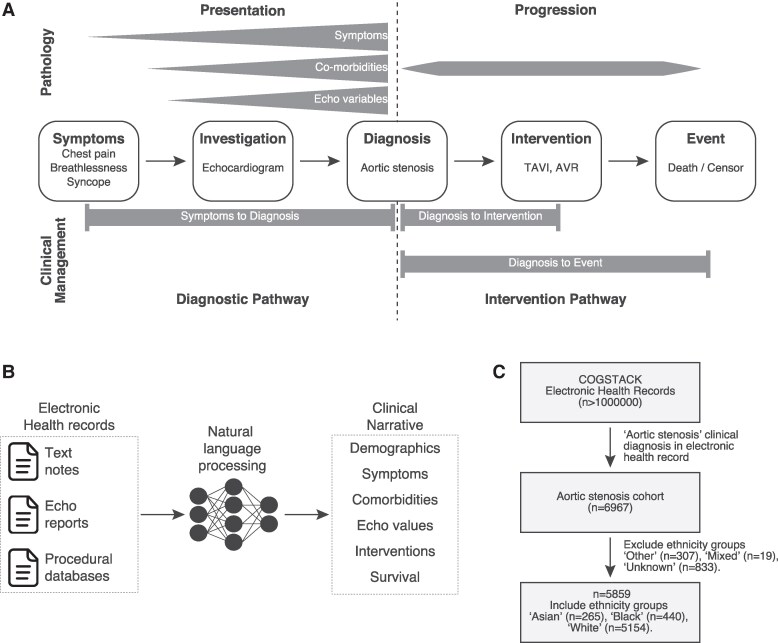
Cohort overview. (*A*) Analysis framework to examine race and ethnicity differences in aortic stenosis pathology and clinical pathways. (*B*) Electronic health record data-types. (*C*) CONSORT diagram of patient inclusion and exclusion criteria.

Here, we characterize racial and ethnic biases at each point in the journey of patients with AS (*Figure 1A*). We apply a natural language processing (NLP)-based artificial intelligence (AI) framework to an electronic database (*Figure 1B*). We conduct a retrospective study using longitudinal electronic health record (EHR) data from a large cohort of patients with AS at a tertiary cardiology hospital (*Figure 1C*). In the research setting, NLP has shown to be accurate at both diagnosing cardiac conditions from free-text notes^10^ and interpreting unstructured echocardiogram reports.^11^ We control for the effects of socioeconomic deprivation by conducting the study within a universal healthcare system that is free at the point-of-care and explicitly adjusting for indices of multiple deprivation. Consequently, we prioritize targets for the most effective healthcare interventions to overcome racial and ethnic biases in AS.

## Methods

This project operated under London South-East Research Ethics Committee approval (18/LO/2048) granted to the King’s Electronic Records Research Interface, which did not require written informed patient consent. This study complies with the Declaration of Helsinki. K.O. had full access to all data in the study and accepts responsibility for its integrity and the data analysis.

### Study cohort

Electronic health records were obtained from King’s College Hospital NHS Foundation Trust (KCH), a multi-site tertiary care hospital. The clinical diagnosis of AS was identified using NLP (an AI technology that allows computers to interpret and understand human language) to extract the SNOMED-CT concept Aortic Valve Stenosis (concept ID: 60573004) and 23 child concepts (see Supplementary material online, *Table S1*). The CogStack NLP pipeline allowed us to capture all mentions of the term ‘aortic valve stenosis’ in the EHR, including the acronym ‘AS’ and synonyms such as ‘aortic stenosis’. Natural language processing identifies mentions of AS from the entire EHR, including clinical notes, discharge summaries, referral letters, medical reports, and other text-based portions. Contextual filters were applied to exclude negations, e.g. patient does not have AS, hypothetical mentions, and mentions referring to individuals other than the patient, e.g. family history of AS, to ensure only confirmed diagnoses are captured. A clinical diagnosis of AS required at least two mentions of AS; these patients were designated to the NLP-derived AS cohort. We included patients with an AS diagnosis between 1 January 2010 and 31 December 2020, with follow-up to 23 December 2022 which is used as the censorship date for survival analyses. We excluded patients under the age of 18 years and those with self-ascribed ethnicity recorded as ‘mixed’, ‘other’, or ‘unknown’ (see Supplementary material online, *Table S2*).

### Data processing

The data (demographic, discharge summaries, clinical notes, TTE reports) were retrieved and analysed in near real-time from the structured (e.g. laboratory observations) and unstructured (free-text documents) components of the EHR using a variety of well-validated NLP informatics tools belonging to the open-source CogStack ecosystem,^12^ MedCAT,^13^ and MedCATTrainer^14^ deployed at KCH. To facilitate effective and efficient search of clinical concepts in the unstructured data MedCAT is employed to annotate the text documents for all SNOMED concepts using a machine learning approach. It is trained in both supervised and unsupervised fashion to achieve good accuracy for the detection of clinical concepts (F1 score > 0.9).^12^ MedCAT uses a concept disambiguation algorithm with word vector contexts and a Bidirectional Long Short-Term Memory model to contextualize clinical information. MedCAT produced unsupervised annotations for all SNOMED-CT concepts under parent terms Clinical Finding, Disorder, Organism, and Event with disambiguation, pre-trained on MIMIC-III.^15^ Further supervised training improved detection of annotations and meta-annotations such as experiencer, negation, and temporality with MedCATTrainer. Performance of the MedCAT NLP pipeline for disorders mentioned in the text has been evaluated on more than 5600 annotations for >265 documents by a domain expert. To assess NLP retrieval bias, 300 patients with orthogonally confirmed AS were randomly sampled, and then, their clinical notes were processed via NLP, showing near-perfect performance across race and ethnicity groups (see Supplementary material online, *Table S3*).

### Study variables

The primary outcomes were symptom and comorbidity burden at AS diagnosis, time from first symptom to AS diagnosis, time from severe AS diagnosis to valvular intervention, and all-cause mortality. Independent variables used in our analysis included race and ethnicity, age, sex, socioeconomic status, AS severity, symptoms, comorbidities, TTE variables, valvular interventions, and survival outcomes. Categories for race and ethnicity were self-reported by patients and extracted from the structured EHR along with age and sex. Socioeconomic deprivation was determined from the Index of Multiple Deprivation (IMD) score from the English Indices of Deprivation 2019 report. The IMD is the government’s official measure of relative deprivation, calculated from 39 indices of socioeconomic status. Neighbourhoods are ranked from most to least deprived.^16^ Postcodes assigned patients to an IMD quintile, with Quintile 5 being assigned to patients residing in the most deprived 20% of neighbourhoods. Aortic stenosis severity was extracted from both echocardiogram reports and clinical notes. Within the echocardiogram reporting software at KCH, Xcelera, the written aspect of the report consists of both free-text and pre-coded sentences, e.g. ‘No haemodynamically significant valvular aortic stenosis’. We used text matching to accurately extract AS severity from these pre-coded sentences, which are handled in the same way as free-text from an NLP perspective. If no data were available in the echocardiogram report, data from the clinical notes were used. In the clinical notes, where AS was identified by MedCAT we searched for the terms ‘mild’/‘mild to moderate’/‘moderate’/‘moderate to severe’/‘severe’ immediately before the AS mentions. We grouped together ‘mild’ and ‘mild to moderate’ as a single mild AS group, with ‘moderate’ and ‘moderate to severe’ as a single moderate AS group. We were able to identify AS severity in 91% of the patients who have an echocardiogram report using text matching, and the results were validated using echocardiogram parameters. Kaplan–Meier analysis was performed to confirm the expected stratification of patient survival based on mild, moderate, and severe disease at 1-year follow-up (see Supplementary material online, *Figure S1A*). Using MedCAT, SNOMED-CT concepts were extracted from clinical notes of patients with AS, including cardiac symptoms (chest pain, breathlessness, palpitations, dizziness, presyncope, and syncope), cardiovascular comorbidities (myocardial infarction, coronary arteriosclerosis, cerebrovascular accident, transient ischaemic attack, peripheral vascular disease, aortic dissection, and aortic aneurysm), and non-cardiovascular comorbidities (Type II diabetes, hypertension, chronic obstructive lung disease, and chronic kidney disease). Structured and unstructured portions of the TTE reports were also used to extract echocardiogram parameters including left ventricular ejection fraction (LVEF), peak velocity, mean gradient, valve area, and velocity ratio. The values from the TTE report with the closest date to the date of AS diagnosis were used to analyse echocardiogram differences at AS presentation. Valvular interventions were also extracted from the EHR, accompanied by manual validation using independent procedural databases demonstrating sensitivity to identify SAVR as 95% and TAVI as 96% (see Supplementary material online, *Table S4*). To mitigate the confounding impact of the COVID-19 pandemic on valvular intervention rates, TAVI and SAVR data were accessed from a shortened timeframe (2010–19). For a small number of patients receiving both SAVR and TAVI procedures (*n* = 12), the earlier procedure was used when stratifying for survival analyses. The date of diagnosis was defined as the first mention of ‘AS’ in the EHR. The date of death was extracted from the EHR, and this, or the last date of the data capture period, was used to calculate survival times.

#### Unselected-echo cohort

For our unselected-echo cohort, we sourced the complete echocardiogram scan database for King’s College Hospital for the time period of the study (*n* = 82 368 patients with 152 013 unique scans). These were filtered for scans reporting an AV *V*_max_ value (*n* = 72 107 patients with 119 759 unique scans), to enable assessment of AS. Lastly, patients were filtered based on self-reported race and ethnicity (*n* = 58 519 patients with 101 011 unique scans) to match with the NLP-derived cohort. Objective diagnosis of AS was made according to BSE guidelines.^17^ The presence of AS on transthoracic echocardiography was defined by aortic valve maximal velocity. Severe AS was classified as AV *V*_max_ ≥ 4 m/s or an AV mean pressure gradient (PG) ≥ 40 mmHg. Moderate AS was classified as an AV *V*_max_ 3.0–3.9 m/s. Lastly, mild AS was classified by an AV *V*_max_ 2.5–2.9 m/s.

### Statistical analysis

Continuous variables were summarized as mean ± standard deviation (or SEM where appropriate) and categorical variables as counts with proportions. Analyses were adjusted as stated for patient age at diagnosis, sex, AS severity at diagnosis, and socioeconomic deprivation quintile (either ‘most deprived’ or ‘other’). Logistic regression calculated odds ratios for being symptomatic or harbouring comorbidities at AS diagnosis. Kruskal–Wallis test was used to compare distributions of echocardiogram values. Linear regression was used to calculate model coefficients for time from symptom to AS diagnosis or time from severe AS diagnosis to valvular intervention. Survival curves were plotted using Kaplan–Meier estimates, with log-rank tests used to assess significantly different survival times. Cox regression was used to calculate hazard ratios (HRs) for time from AS diagnosis to all-cause death or censoring. Schoenfeld residuals were plotted to evaluate the proportional hazard assumption. Statistical analyses were performed using R (version 4.3). All statistical tests were two-sided, unless otherwise stated, with *P*-values <0.05 designated statistically significant.

## Results

### Patient characteristics

We applied a NLP search to >1 000 000 individual patient records (see Methods), identifying 6967 patients with a clinical diagnosis of AS (*Figure 1C*). Self-reported race and ethnicity codes were used to identify sufficiently large groups for comparative analysis (see Supplementary material online, *Figure S1B*). Overall, 5859 patients were included for analysis, composed of patients with an ethnicity label of White (*n* = 5154), Black (*n* = 440), or Asian (*n* = 265).

Examining baseline characteristics, significant differences were observed in age, sex, and socioeconomic status between race and ethnicity groups (*Table 1*). The average age at AS diagnosis for White patients (77.2 years) and Asian patients (77.2 years) was higher than for Black patients (74.8 years). The majority of Black patients were female (60.7%), with the opposite seen in both Asian (37.7%) and White (45.3%) patients. A higher proportion of Black patients (30.4%) were classified within the most deprived IMD quintile compared with White (11.8%) or Asian patients (10.5%, Supplementary material online, *Figure S1C*).

**Table 1:** Baseline characteristics

	All	Asian	Black	White	*P*-value
***Patient demographics***					
Number of patients	5859	265 (4.5%)	440 (7.5%)	5154 (88.0%)	—
Age, years	76.8 (12)	72.2 (13.1)	74.8 (13.1)	77.2 (11.8)	<0.001
Female	2703 (46.1%)	100 (37.7%)	267 (60.7%)	2336 (45.3%)	<0.001
IMD most deprived quintile	587 (13.3%)	21 (10.5%)	114 (30.5%)	452 (11.8%)	<0.001
***AS severity***					
Mild	1667 (28.5%)	93 (35.1%)	164 (37.3%)	1410 (27.4%)	
Moderate	1404 (24.0%)	66 (24.9%)	88 (20.0%)	1250 (24.3%)	
Severe	1914 (32.7%)	58 (21.9%)	81 (18.4%)	1775 (34.4%)	
Unknown	874 (14.9%)	48 (18.1%)	107 (24.3%)	719 (14.0%)	
***AS symptoms***					
Chest pain	1288 (22%)	79 (29.8%)	164 (37.3%)	1045 (20.3%)	<0.001
Breathlessness	1959 (33.4%)	103 (38.9%)	206 (46.8%)	1650 (32%)	<0.001
Palpitations	307 (5.2%)	16 (6%)	40 (9.1%)	251 (4.9%)	<0.001
Dizziness	623 (10.6%)	40 (15.1%)	87 (19.8%)	496 (9.6%)	<0.001
Presyncope	75 (1.3%)	4 (1.5%)	4 (0.9%)	67 (1.3%)	n.s.
Syncope	321 (5.5%)	13 (4.9%)	50 (11.4%)	258 (5%)	<0.001
Symptomatic at AS diagnosis (any)	2563 (43.7%)	121 (45.7%)	273 (62.0%)	2169 (42.1%)	—
***AS comorbidities***					
Body mass index (kg/m^2^)	27.9 ± 7.0	26.5 ± 6.6	30 ± 8.3	27.8 ± 6.8	<0.001
Myocardial infarction	485 (8.3%)	30 (11.3%)	50 (11.4%)	405 (7.9%)	<0.01
Coronary arteriosclerosis	399 (6.8%)	26 (9.8%)	44 (10%)	329 (6.4%)	<0.01
Hypertension	2326 (39.7%)	106 (40%)	289 (65.7%)	1931 (37.5%)	<0.001
Peripheral vascular disease	156 (2.7%)	11 (4.2%)	18 (4.1%)	127 (2.5%)	<0.05
Stroke/TIA	820 (14%)	38 (14.3%)	82 (18.6%)	700 (13.6%)	<0.05
Chronic obstructive pulmonary disease	542 (9.3%)	16 (6%)	43 (9.8%)	483 (9.4%)	n.s.
Chronic kidney disease	415 (7.1%)	24 (9.1%)	60 (13.6%)	331 (6.4%)	<0.001
Diabetes mellitus (type 2)	806 (13.8%)	71 (26.8%)	129 (29.3%)	606 (11.8%)	<0.001
Comorbid at AS diagnosis (any)	2915 (49.8%)	132 (49.8%)	321 (73.0%)	2462 (47.8%)	—

Values are *n* (%) or mean ± standard deviation. Baseline characteristics were compared using a Pearson χ^2^ test for categorical variables and a Kruskal–Wallis test for continuous variables.

### Aortic stenosis presentation

**Figure 2:**
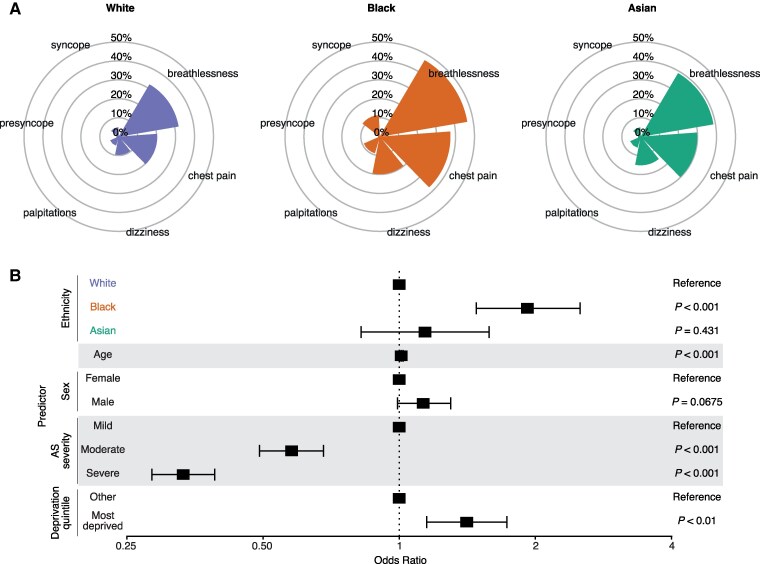
Aortic stenosis presentation. (*A*) Radar plots showing the prevalence of cardiac symptoms at aortic stenosis diagnosis, plotted separately by ethnicity. The symptoms categories are chest pain, breathlessness, palpitations, dizziness, presyncope, and syncope. The percentages for each symptom are displayed within the corresponding radar slice. (*B*) Forest plot showing the adjusted odds ratios for presenting with any cardiac symptoms at the point of diagnosis for each ethnicity group. A logistic regression model is adjusted for age, sex, aortic stenosis disease severity, and socioeconomic deprivation. Error bars represent the limits of the 95% confidence interval for the odds ratio.

We explored racial and ethnic differences in the pattern and burden of cardiac symptoms at AS diagnosis. Breathlessness and chest pain were the most reported cardiac symptoms across all ethnicities (*Table 1*, *Figure 2A*). A greater proportion of Black patients were symptomatic at diagnosis (62.0%) than White (42.1%) or Asian (45.7%) patients. Further, in a logistic regression model adjusted for age, sex, AS disease severity, and socioeconomic deprivation, Black patients were significantly more likely to exhibit symptoms at AS diagnosis compared with White patients {adjusted odds ratio [aOR] = 1.92, [95% confidence interval (CI) = 1.48–2.51]}, *P* < 0.001, *Figure 2B*).

Quantifying the frequency of comorbidities at AS diagnosis, we observed significant racial and ethnic differences in a range of cardiometabolic comorbidities, most notably hypertension (*Table 1*, Supplementary material online, *Figure S2A*). A higher proportion of Black patients (73.0%) were comorbid at AS diagnosis than White (47.8%) or Asian patients (49.8%, Supplementary material online, *Figure S2B*). Black patients were significantly more likely to harbour comorbidities at AS diagnosis compared with White patients [aOR = 2.66 (95% CI = 1.98–3.61), *P* < 0.001, Supplementary material online, *Figure S2C*] in a logistic regression model, adjusted for age, sex, AS disease severity, and socioeconomic deprivation.

At AS diagnosis, White patients were most commonly diagnosed with severe disease (40.0%), whereas only 24.3% of Black patients and 26.7% of Asian patients were diagnosed with severe AS (*Table 1*, Supplementary material online, *Figure S3A*). Higher rates of bicuspid aortic valves were seen in White (10.7%) and Asian (10.9%) patients than in Black (7.0%) patients (see Supplementary material online, *Figure S3A*). Examining echocardiographic parameters of left ventricular (LV) structure, Black patients displayed increased LV thickness and higher LVEFs relative to White patients (see *Table 2*, Supplementary material online, *Figure S3B*).

**Table 2:** Echocardiogram values at time of diagnosis

	All	Asian	Black	White	*P*-value
AVA (cm^2^)	1.16 ± 0.494	1.19 ± 0.443	1.34 ± 0.51	1.14 ± 0.491	<0.001
AVA indexed (cm^2^/m^2^)	0.59 ± 0.196	0.68 ± 0.225	0.70 ± 0.247	0.58 ± 0.188	<0.001
AV *V*_max_ (cm/s)	331 ± 109	300 ± 108	293 ± 103	337 ± 109	<0.001
AV mean PG (mmHg)	27.6 ± 17.9	23.1 ± 16.9	21.6 ± 15.3	28.4 ± 18.1	<0.001
LVEF (%)	66.6 ± 15.3	68 ± 16.6	70.1 ± 14.1	66.1 ± 15.3	<0.001
LVPWd (mm)	1.18 ± 0.222	1.10 ± 0.2	1.25 ± 0.237	1.17 ± 0.219	<0.001
IVSd (mm)	1.28 ± 0.246	1.19 ± 0.229	1.34 ± 0.271	1.28 ± 0.243	<0.001
LVIDd (cm)	4.62 ± 0.775	4.48 ± 0.731	4.44 ± 0.76	4.65 ± 0.777	<0.001
LVIDs (cm)	3.19 ± 0.856	3.09 ± 0.883	2.93 ± 0.805	3.22 ± 0.855	<0.001
LV mass (g)	218 ± 72.1	187 ± 55.3	223 ± 82.3	219 ± 71.4	<0.001
LA volume (mL)	74.7 ± 32.6	62.9 ± 33.1	72.0 ± 26.3	75.9 ± 33	<0.001
TR max velocity (m/s)	266 ± 51	259 ± 47	269 ± 50.9	267 ± 51.2	n.s.
Relative wall thickness	0.275 ± 0.0764	0.266 ± 0.0833	0.302 ± 0.0867	0.272 ± 0.0743	<0.001
Bicuspid aortic valve	612 (10.4%)	29 (10.9%)	31 (7.0%)	552 (10.7%)	—

Values are mean ± standard deviation.

### Aortic stenosis clinical management

**Figure 3:**
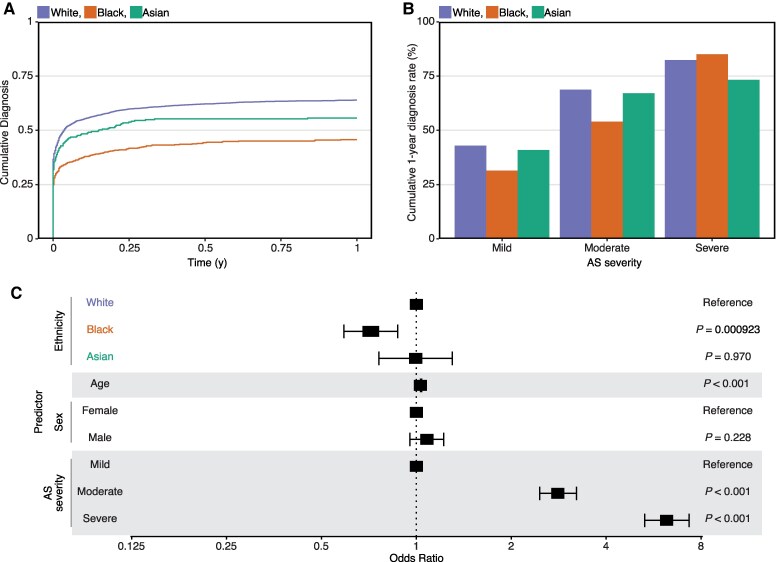
Aortic stenosis diagnosis. (*A*) Line plot showing cumulative percentage of aortic stenosis clinical diagnosis in the first year after positive echocardiogram findings, stratified by race and ethnicity. (*B*) One-year rate (percentage) of aortic stenosis clinical diagnosis stratified by race and ethnicity. (*C*) Forest plot showing the adjusted odds ratios for aortic stenosis clinical diagnosis in the first year after positive echocardiogram findings for each ethnicity group. A logistic regression model is adjusted for age, sex, and aortic stenosis disease severity. Error bars represent the limits of the 95% confidence interval for the odds ratio.

We calculated the time interval between the first positive echocardiogram scan with objective evidence of AS and the first clinical documentation of an AS diagnosis (including mentions in the echocardiogram report, clinic letters, and ward round entries). For this analysis, we linked the NLP-derived AS cohort (*n* = 5859) with the complete database of echocardiogram scans for unselected indications (*n* = 58 519 patients with 101 011 unique scans) sourced from the same hospital over the time period for the study (see Methods). The rate of clinical diagnosis of AS within 1 year of a positive echocardiogram scan was lower for Black patients (45.8%) than for White (63.9%) or Asian (55.7%) patients (*Figure 3A*). Black patients with echocardiographic evidence of mild or moderate AS received clinical diagnoses at lower rates than White patients (*Figure 3B*), with a difference of 11.5% for mild AS (1-year cumulative diagnosis rates for mild AS: White = 42.9%, Black = 31.4%, Asian = 40.9%) and 14.7% for moderate AS (1-year cumulative diagnosis rates for moderate AS: White = 68.7%, Black = 54.0%, and Asian = 67.1%). In severe AS, there was no difference observed between Black and White patients (*Figure 3C*); however, the diagnosis rate was lower for Asian patients (1-year cumulative diagnosis rates for severe AS: White = 82.4%, Black = 85.0%, and Asian = 73.2%). A logistic regression model confirmed the longer echocardiogram-to-diagnosis time for Black patients compared with White patients was robust to the effects of patient age, sex, and AS disease severity [coefficient = 0.717 (95% CI = 0.589–0.873), *P* < 0.001, *Figure 3C*].

Utilizing NLP-derived symptom data, we further calculated time intervals between the first mention of a cardiac symptom and AS diagnosis. Analysing breathlessness, the commonest symptom experienced at AS diagnosis in this cohort (*Table 1*), the mean symptom-to-diagnosis time for Black patients was 2.93 years (SEM = 2.65–3.21), compared with 2.62 years (2.27–2.96) for Asian patients and 2.12 years (2.03–2.21) for White patients (see Supplementary material online, *Figure S4A*). A linear regression model confirmed the longer symptom-to-diagnosis time for Black patients compared with White patients was robust to the effects of patient age, sex, AS disease severity, and socioeconomic deprivation [coefficient = 2.09 (95% CI = 1.40–3.12), *P* < 0.001, Supplementary material online, *Figure S4A*]. For chest pain, the mean symptom-to-diagnosis time for Asian patients [mean = 2.70 years, (SEM = 2.37–3.03)] was longer than for Black [2.37 years, (2.14–2.60)] or White [1.69 years, (1.62–1.76)] patients (see Supplementary material online, *Figure S4B*). This result was also significant in an adjusted linear regression model [coefficient = 2.93 (95% CI = 1.49–5.78), *P* < 0.01, Supplementary material online, *Figure S4B*]. There were insufficient mentions of other cardiac symptoms (palpitations, dizziness, presyncope, and syncope) for Black and Asian patients to calculate symptom-to-diagnosis time intervals.

**Figure 4:**
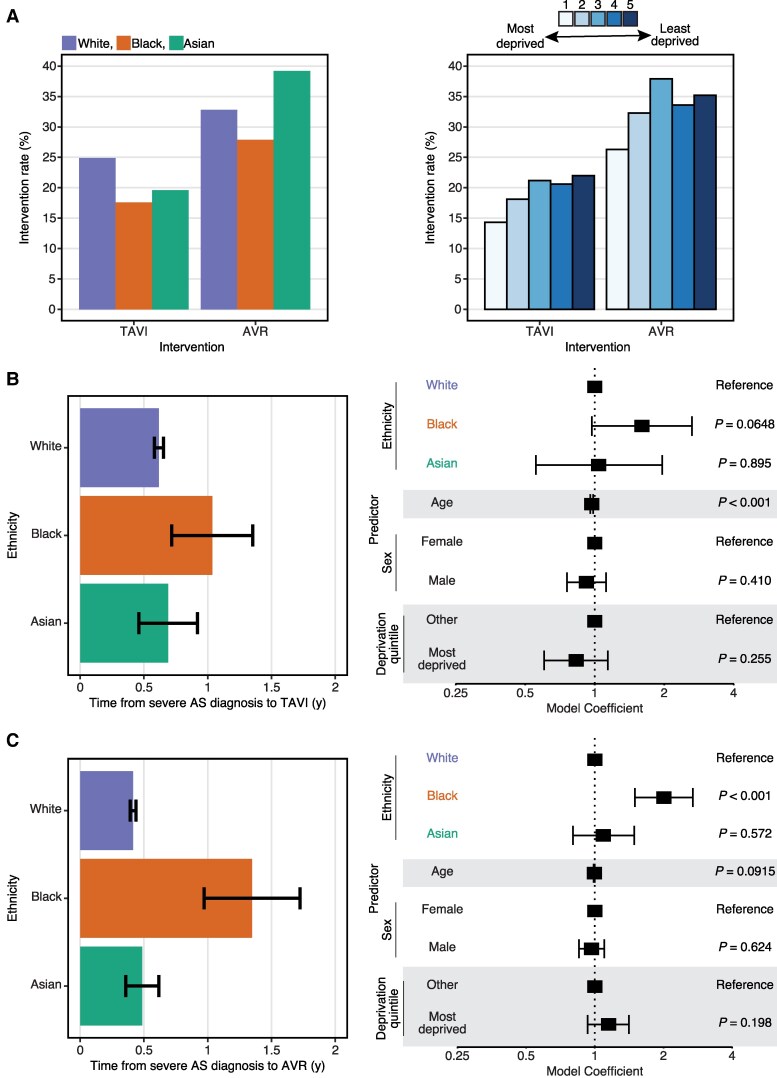
Aortic stenosis intervention. (*A*) Bar plots showing the percentage of patients diagnosed with severe aortic stenosis receiving an intervention (transcatheter aortic valve implantation or surgical aortic valve replacement), stratified by ethnicity (left) or by deprivation quintile (right). (*B*) Time between severe aortic stenosis diagnosis and transcatheter aortic valve implantation, stratified by ethnicity. Bar plot (left) shows mean time difference. Forest plot (right) shows coefficients for a linear regression model adjusted for age, sex, and socioeconomic deprivation. (*C*) Time between severe aortic stenosis diagnosis and surgical aortic valve replacement, stratified by ethnicity. Bar plot (left) shows mean time difference. Forest plot (right) shows coefficients for a linear regression model adjusted for age, sex, and socioeconomic deprivation. Error bars for bar plots represent the SEM. Error bars for forest plots represent the limits of the 95% confidence interval for the model coefficient.

We performed a subgroup analysis of patients diagnosed with severe AS to identify race and ethnicity differences in the frequency of valvular interventions (TAVI or SAVR). A higher proportion of White patients (24.9%) had a TAVI procedure than Black (17.6%) or Asian (19.6%) patients (*Figure 4A*). Similarly, higher proportions of White (32.8%) and Asian (39.2%) patients had a SAVR procedure, with a lower proportion received by Black (27.9%) patients (*Figure 4A*). We also examined differences in valvular intervention frequency according to socioeconomic status (*Figure 4A*).

We next calculated time intervals from the diagnosis of severe AS to valvular intervention in this patient subgroup. For TAVI procedures, the mean diagnosis-to-intervention time for White patients was 0.62 years (SEM = 0.58–0.65), compared with 1.03 years (0.72–1.35) for Black patients and 0.69 years (0.46–0.92) for Asian patients (*Figure 4B*). In a linear regression model controlling for age, sex, and socioeconomic deprivation, ethnicity was not significantly associated with time to TAVI (*Figure 4B*). For SAVR procedures, the mean diagnosis-to-intervention time for Black patients [mean = 1.35 years (SEM = 0.97–1.72)] was longer than for Asian [0.49 years (0.36–0.62)] or White [0.41 years (0.39–0.44)] patients (*Figure 4C*). The longer time to SAVR in Black patients compared with White patients was statistically significant in a linear regression model adjusting for age, sex, and socioeconomic deprivation [adjusted model coefficient = 2.01 (95% CI = 1.50–2.69), *P* < 0.001, *Figure 4C*].

### Aortic stenosis mortality

To explore the survival association of race and ethnicity in our cohort, we examined 5-year mortality post-diagnosis. This revealed no differences in survival times between patients of different ethnicities (see Supplementary material online, *Figure S5A*). Multivariate Cox analysis confirmed no relationship between ethnicity and survival outcomes in the full cohort and also revealed that increasing age, male sex, and socioeconomic deprivation were significantly associated with increased mortality (see Supplementary material online, *Figure S5B*).

**Figure 5:**
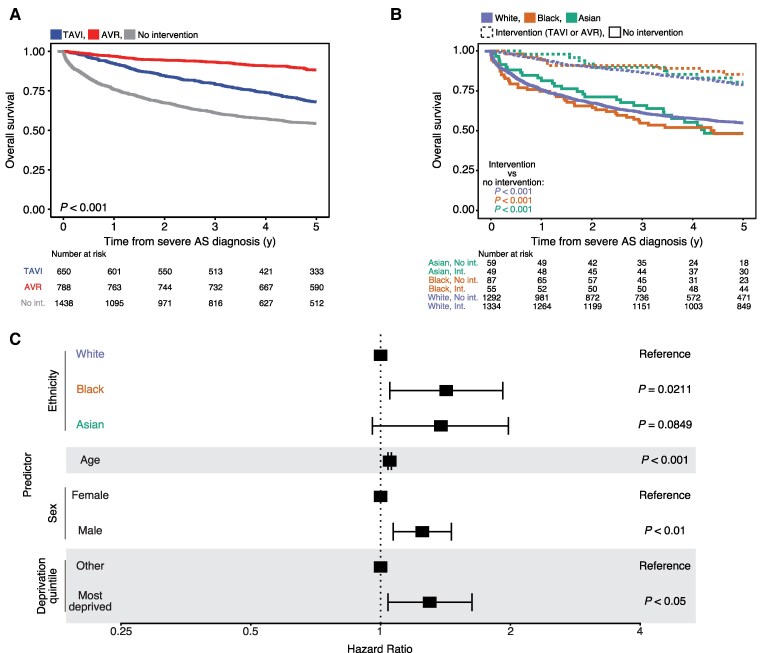
Aortic stenosis mortality. (*A*) Kaplan–Meier plot showing overall survival outcomes from severe aortic stenosis diagnosis stratified by intervention. (*B*) Kaplan–Meier plot showing overall survival outcomes from diagnosis of severe aortic stenosis stratified by ethnicity and intervention status. (*C*) Forest plot showing hazard ratios for multivariate Cox analysis of overall survival outcomes from diagnosis of severe aortic stenosis stratified by ethnicity, age, sex, and socioeconomic deprivation.

Next, we assessed survival times in patients diagnosed with severe AS, where valvular interventions may be indicated. Examining survival outcomes by intervention status, TAVI or SAVR procedures were associated with a significant survival benefit (*Figure 5A*, *P* < 0.001). Subgrouping patients by race and ethnicity, any valvular intervention (TAVI or SAVR) was associated with a significant survival benefit, indicating that intervention was life-prolonging regardless of ethnicity (*Figure 5B*).

Lastly, we performed a multivariate Cox analysis of patients with severe AS. It was not possible to formally integrate intervention status (TAVI or SAVR) in the Cox model as the proportional hazards assumption was violated (Schoenfeld residuals shown in Supplementary material online, *Figure S6*). Black patients experienced higher rates of mortality [*Figure 5C*, HR = 1.42 (95% CI = 1.05–1.92), *P* = 0.0211].

### Aortic stenosis prevalence

**Figure 6:**
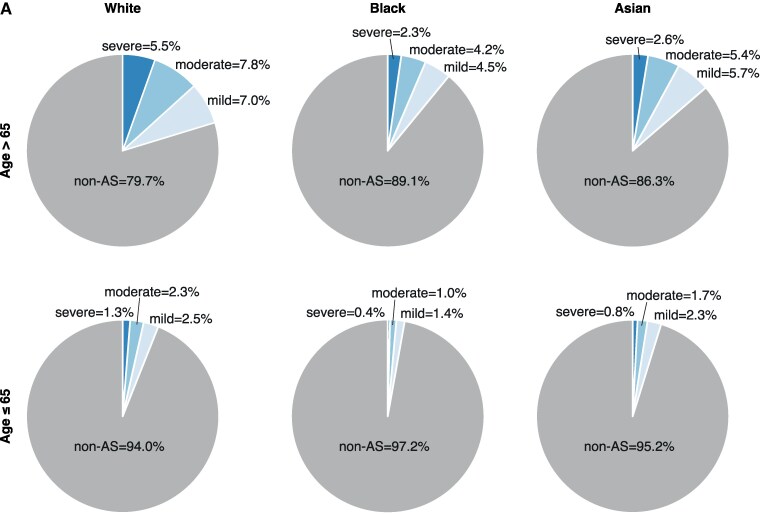
Aortic stenosis prevalence. (*A*) Pie charts showing aortic stenosis prevalence by race and ethnicity in >100 000 echocardiograms for unselected indication. Top row shows data for people over the age of 65 years; bottom row shows data for people below the age of 65 years.

We analysed the prevalence of AS in the natural population, stratified by race and ethnicity, using >100 000 echocardiograms for unselected indication (see Methods, Supplementary material online, *Table S5*). In patients above the age of 65 years, AS was most prevalent in White patients (20.3%) compared with Black (10.9%) and Asian (13.7%) patients (*Figure 6*). A similar pattern was seen among patients below the age of 65 years (AS rates: Asian = 4.8%, Black = 2.8%, and White = 6.0%).

## Discussion

In this single-centre, retrospective, observational study, we curated the EHRs of ∼7000 patients with a clinical diagnosis of AS to investigate whether race and ethnicity are associated with differences in clinical presentation, management, and outcomes. By conducting this study at a central London hospital and extracting indices of socioeconomic deprivation, we evaluated whether racial and ethnic disparities persist in a universal healthcare system and explicitly adjusting for the effects of socioeconomic deprivation.

Black patients are more likely to present at younger age, be female, and harbour comorbidities at AS diagnosis compared with White patients, consistent with the existing literature.^18^ Natural language processing retrieval suggested that 44% of patients were symptomatic at AS diagnosis, which is in line with prior data indicating that one-third to half of patients with AS are asymptomatic, even with severe disease.^19,20^ Interestingly, we make the novel observation that a higher proportion of Black patients exhibit cardiac symptoms at AS diagnosis. This finding is striking given previous work reporting Black patients are less likely to report breathlessness symptoms and more likely to misattribute chest pain to gastrointestinal causes.^21,22^ Additional contributing factors may include patient-related aspects such as health awareness and healthcare mistrust, as well as healthcare system-related factors such as cultural and language barriers.^9^

We also observed racial and ethnic variations in LV echocardiographic parameters of patients with severe AS. Compared with White patients, Black patients are more likely to have larger, thicker ventricles, and higher LVEF values. These differences may be attributed to ethnicity-specific patterns of AS-related LV remodelling or to the higher prevalence of hypertension in Black patients relative to White patients. The combination of these factors could compound to further impair LV compliance and elevate filling pressures,^23^ which may explain why Black patients more frequently reported breathlessness as a symptom. Future research including time-series echocardiogram analysis could assess whether observed LV structural changes are static, reflecting a chronic hypertensive state, or are progressive due to LV remodelling with increasing AS severity.

A previous EHR study based on ICD codes found higher AS diagnosis rates in the presence of a positive echocardiogram scan for White individuals than for Black individuals.^6^ Here, we validate this result, showing that Black patients with evidence of mild or moderate disease on echocardiography are less likely to receive clinical diagnosis of AS than White or Asian patients. Natural language processing retrieval of AS diagnosis potentially mitigated the impact of structural biases in ICD coding systems and the undercoding of AS as a primary or secondary diagnosis among patients with multiple comorbidities.^24^ A timely clinical diagnosis is critical, as it serves as the gateway to further clinical evaluation, including regular valve surveillance in a structural cardiology clinic and consideration for valvular intervention. Establishing a diagnosis at this stage is particularly crucial as a recent multi-national meta-analysis reveals moderate AS to be associated with significant mortality and corresponding lifetime loss, even in the presence of a normal ejection fraction.^25^ Failure to diagnose moderate AS in Black patients therefore represents a missed opportunity for early management, and a healthcare intervention to decrease the proportion of patients missed following a positive echocardiogram scan would be highly beneficial. Future work may evaluate a real-time EHR-generated prompt, for example flagging an abnormal echocardiogram scan result for clinician review. A similar digital intervention strategy has recently been shown to improve clinician adherence to the prescribing of guideline-directed medical therapy in heart failure clinics.^26^

Exploring valvular interventions in severe AS, Black patients have lower TAVI rates and wait longer for SAVR. Among patients with severe AS—and adjusting for age, sex, and socioeconomic deprivation—Black ethnicity is associated with increased mortality. Notably, once selected for valvular intervention, all race and ethnicity groups experience favourable outcomes with no differences in mortality as previously described.^27,28^ Although race and ethnicity are social constructs with no evidence of differing biological mechanisms,^29^ the association of Black ethnicity with adverse prognosis in severe AS warrants investigation into potential underlying mechanisms, including structural racism. Future work may examine the utility of an EHR-based system to monitor the rates of valvular intervention by race and ethnicity group to address disparities in intervention uptake and downstream mortality outcomes. Additional strategies to actively address the treatment gap may include cultural training for healthcare providers and improved patient educational campaigns.^9^ As a research community, there is a pressing need to increase the representation and reporting of race and ethnicity data in valvular heart disease clinical trials.^30^

The findings of this study are derived from a large, single-centre, retrospective database that uses AI-based methods to identify patients with AS. Observational datasets with long-term follow-up can characterize treatment disparities. However, traditional methods of prospective registry creation have several limitations, including significant human resource, and incompleteness in case record selection stemming from inaccurate clinical coding. Therefore, a strength of the study is the novel use of NLP to extract unstructured, free-text EHR data (e.g. uncoded diagnoses, symptoms, and comorbidities written in the clinical text) for automated cohort generation as we have demonstrated in our recent work.^31^ In addition, AI-based extraction of symptom, comorbidity, and echocardiogram data directly from the clinical record facilitates high-resolution temporal analyses, including the calculation of symptom-to-diagnosis and diagnosis-to-intervention times.

To our knowledge, this is the first comprehensive study examining the effects of race, ethnicity, and socioeconomic deprivation in AS within a universal healthcare system (the UK National Health Service is ‘free at the point of use’). Our findings therefore add to the existing literature of North American studies, where potential payor biases may significantly influence patient and clinician behaviour.

The importance of integrating data on socioeconomic deprivation is indicated by several of our multivariate analyses, as IMD independently associates with being symptomatic and comorbid at diagnosis and with mortality outcomes. However, there are limitations in the use of IMD as a metric of socioeconomic deprivation. Firstly, the choice of domains used to form the composite index is subjective and does not encompass the totality of socioeconomic deprivation. Secondly, it is important to recognize that IMD is a rank of an area’s deprivation compared with other areas within England and so represents an indirect measure of the socioeconomic deprivation experienced at a patient level. For example, an individual can be exposed to deprivation even if living in a relatively less deprived area.

This study has other important limitations. First, our dataset is subject to the same potential biases as any other single-centre retrospective observational study. Our findings are observational in nature, and relationships between variables do not imply causation. Second, the use of EHRs may also introduce bias as it has been noted that physicians’ engagement and patient descriptors may vary in clinic notes by patient race and ethnicity.^32,33^ Third, due to the proximity of London hospitals, it is possible that some patients prior AS investigations or management were not reflected in the EHR accessed for this study. For example, it is possible that patients diagnosed at KCH may have undergone valve replacement elsewhere. However, as KCH is a tertiary cardiology centre with a catchment area of over 1 million patients covering five different London boroughs and receives referrals for patients from a number of Kent hospitals outside London, the proportion of such patients would be small. In future work, we plan to validate and expand upon our findings by analysing EHRs from other hospital sites with the CogStack NLP pipeline deployed. This will provide a more comprehensive perspective and allow for a more robust comparison of data across diverse settings. Fourth, for the unselected-echo cohort, AS severity was based on AV Vmax alone. The use of a singular echocardiographic metric aligns with previous work assembling an AS registry from retrospective EHRs;^6^ however, this may misdiagnose high-output states or low-flow low-gradient AS, so the distributions of these subgroups by race and ethnicity have not been explored. Finally, we acknowledge the unequal representation of racial and ethnic groups, with White patients comprising the majority of the cohort, which may make it difficult to assess the independence of the effects of ethnicities on the outcome measures from those of socioeconomic deprivation, especially in Black or Asian patients. We note that this reflects the race and ethnicity of a real-world AS cohort drawn from a racially and socioeconomically diverse population in South London and that the study inclusion criteria are not biased by ICD coding as for previous work in this area.^4–6^ A further limitation is the use of high-level categories for race and ethnicity. Future research using larger datasets will allow for more granular categorization (e.g. the separation of Asian patients into South Asian and East Asian categories) and the inclusion of categories not studied in this work due to insufficiently large groups for comparative analysis (e.g. Middle Eastern or Hispanic patients) for added global relevance. Lastly, while we have quantified racial and ethnic disparities in AS presentation, diagnosis, intervention, and mortality, it is not possible to attribute causality to specific mechanisms in a retrospective, observational study. For instance, the delayed AS diagnosis observed in Black patients compared with White patients may stem from patient-related factors, such as delayed self-presentation, as Black patients may interpret chest pain differently,^21,22^ or from valve differences, such as Black patients’ tendencies towards higher valve areas and lower valve gradients (*Table 2*).^34^ Alternatively, healthcare-related factors, including potential physician bias in diagnostic reasoning, may contribute, as Black patients are often younger and present with more comorbidities at the time of AS diagnosis. Moreover, the study design only permits analysis of patients who had undergone echocardiography, preventing us from assessing potential biases in referral to echocardiography, which may further amplify the observed disparities. In future, large-scale prospective studies could attribute causality to specific interventions, dissect the upstream influence of healthcare provider referral to echocardiography, as well as determine the true prevalence of AS by race and ethnicity group.

## Conclusions

Applying NLP to routinely collected EHR data enables the detailed characterization of racial and ethnic disparities throughout the clinical journey of patients with AS. Black patients with echocardiographic findings consistent with mild or moderate AS were less likely to receive a clinical diagnosis than White or Asian patients. In patients with severe AS, TAVI and SAVR procedures were both performed at lower rates among Black patients than among White patients, and the mean time to SAVR was longer. In a multivariate analysis of patients with severe AS, which controlled for socioeconomic status in a universal healthcare system, Black patients experienced higher rates of mortality. These data should stimulate targeted healthcare interventions to reduce inequity.

## Supplementary Material

ztaf018_Supplementary_Data

## Data Availability

The data and code underlying this article will be shared on reasonable request to the corresponding author.
