# Supplementary material for: Racial and ethnic disparities in aortic stenosis within a universal healthcare system characterized by natural language processing for targeted intervention
Source: Eur Heart J Digit Health. 2025 Mar 18;6(3):392–403. doi: 10.1093/ehjdh/ztaf018 (PMC12088714; doi:10.1093/ehjdh/ztaf018)
Supplement: ztaf018_Supplementary_Data [file ztaf018_supplementary_data.zip › supplementary_1.pdf]

Supplementary Figure S1

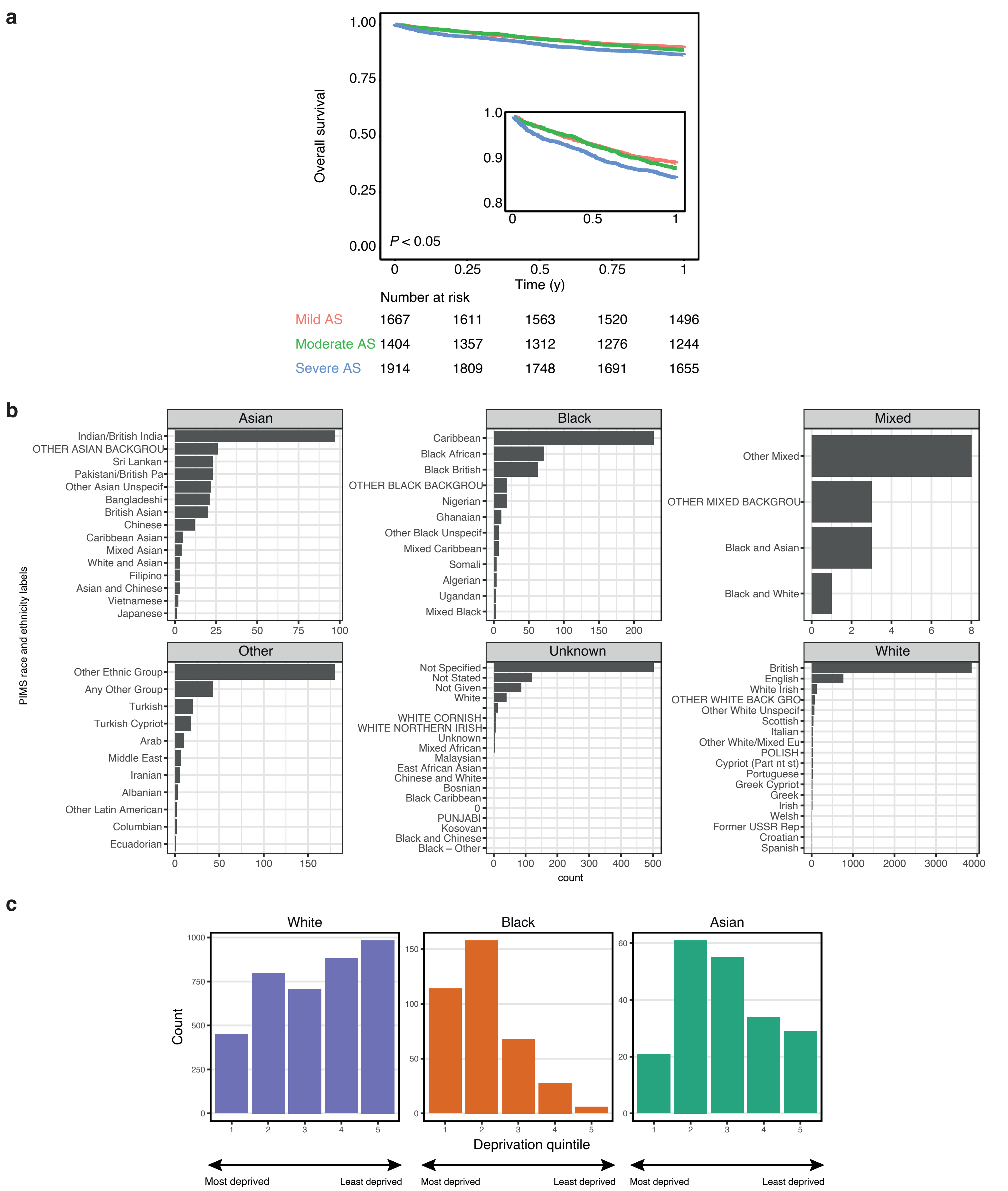

Supplementary Figure S1 | Baseline characteristics

a, Kaplan-Meier curves with log-rank P values are shown for the study cohort stratifed by severity of AS over a 1-year period post-diagnosis.
b, Bar-plots are shown for each race and ethnicity label extracted from the Patient Information Management System (PIMS). Each top-level ethnicity label (i.e. Asian, Black, Mixed, Other, Unknown, White) is plotted as a separate bar-plot, then each individual race-code is shown as a bar.
c, Bar-plots are shown for socioeconomic deprivation as IMD quintiles.
