# Supplementary material for: Racial and ethnic disparities in aortic stenosis within a universal healthcare system characterized by natural language processing for targeted intervention
Source: Eur Heart J Digit Health. 2025 Mar 18;6(3):392–403. doi: 10.1093/ehjdh/ztaf018 (PMC12088714; doi:10.1093/ehjdh/ztaf018)
Supplement: ztaf018_Supplementary_Data [file ztaf018_supplementary_data.zip › supplementary_2.pdf]

Supplementary Figure S2

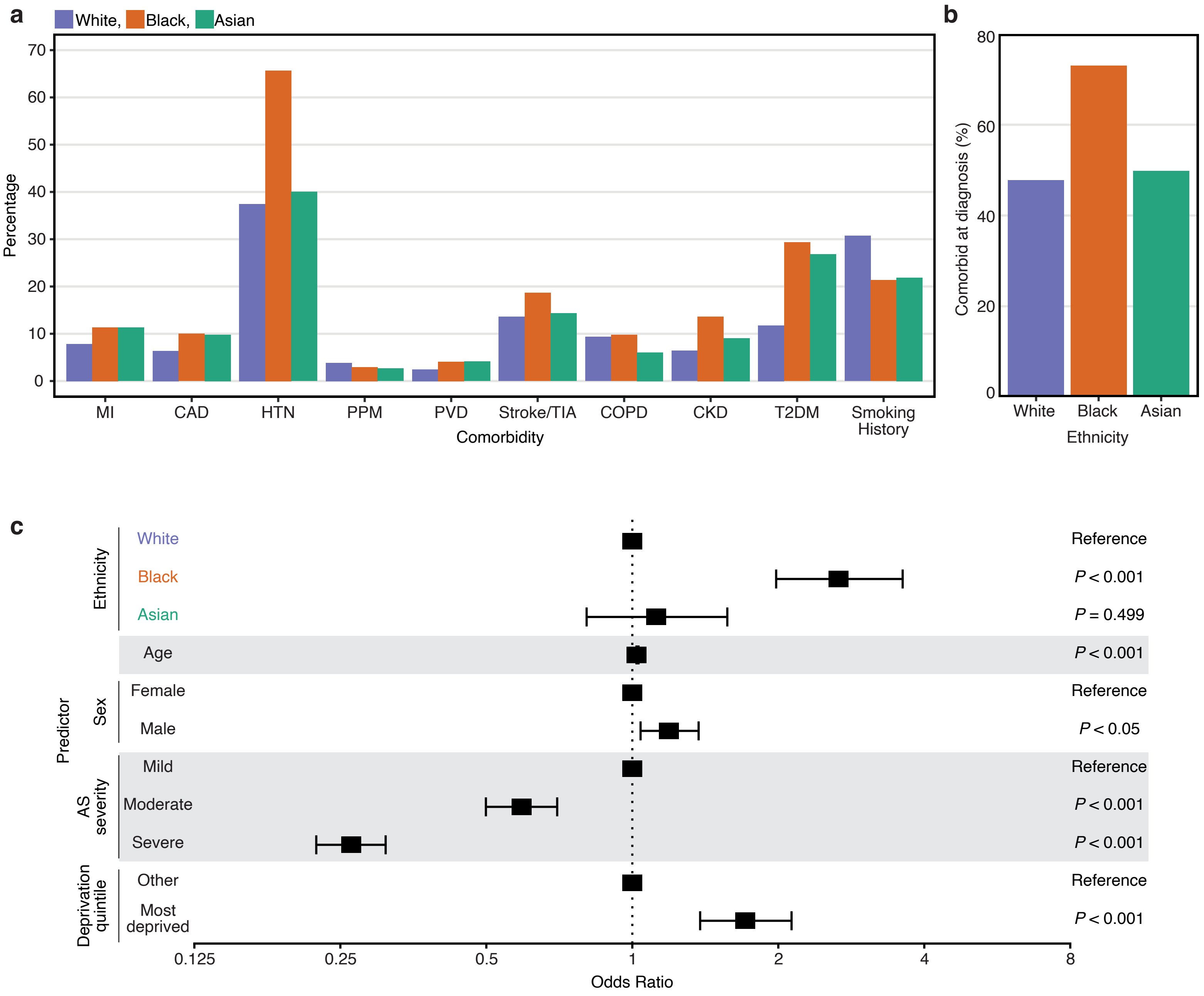

Supplementary Figure S2 | Comorbidities at AS diagnosis

a, Bar plot showing the percentage of patients harbouring comorbidities at AS diagnosis, plotted separately by ethnicity.
b, Bar plot shows the percentage of patients presenting with any comorbidities at AS diagnosis.
c, Forest plot showing the adjusted odds ratios for harbouring any comorbidities at AS diagnosis for each ethnicity group. A logistic regression model is adjusted for age, sex, AS disease severity and socioeconomic deprivation. Error bars represent the limits of the 95% confidence interval for the odds ratio.
