# Supplementary material for: Racial and ethnic disparities in aortic stenosis within a universal healthcare system characterized by natural language processing for targeted intervention
Source: Eur Heart J Digit Health. 2025 Mar 18;6(3):392–403. doi: 10.1093/ehjdh/ztaf018 (PMC12088714; doi:10.1093/ehjdh/ztaf018)
Supplement: ztaf018_Supplementary_Data [file ztaf018_supplementary_data.zip › supplementary_3.pdf]

Supplementary Figure S3

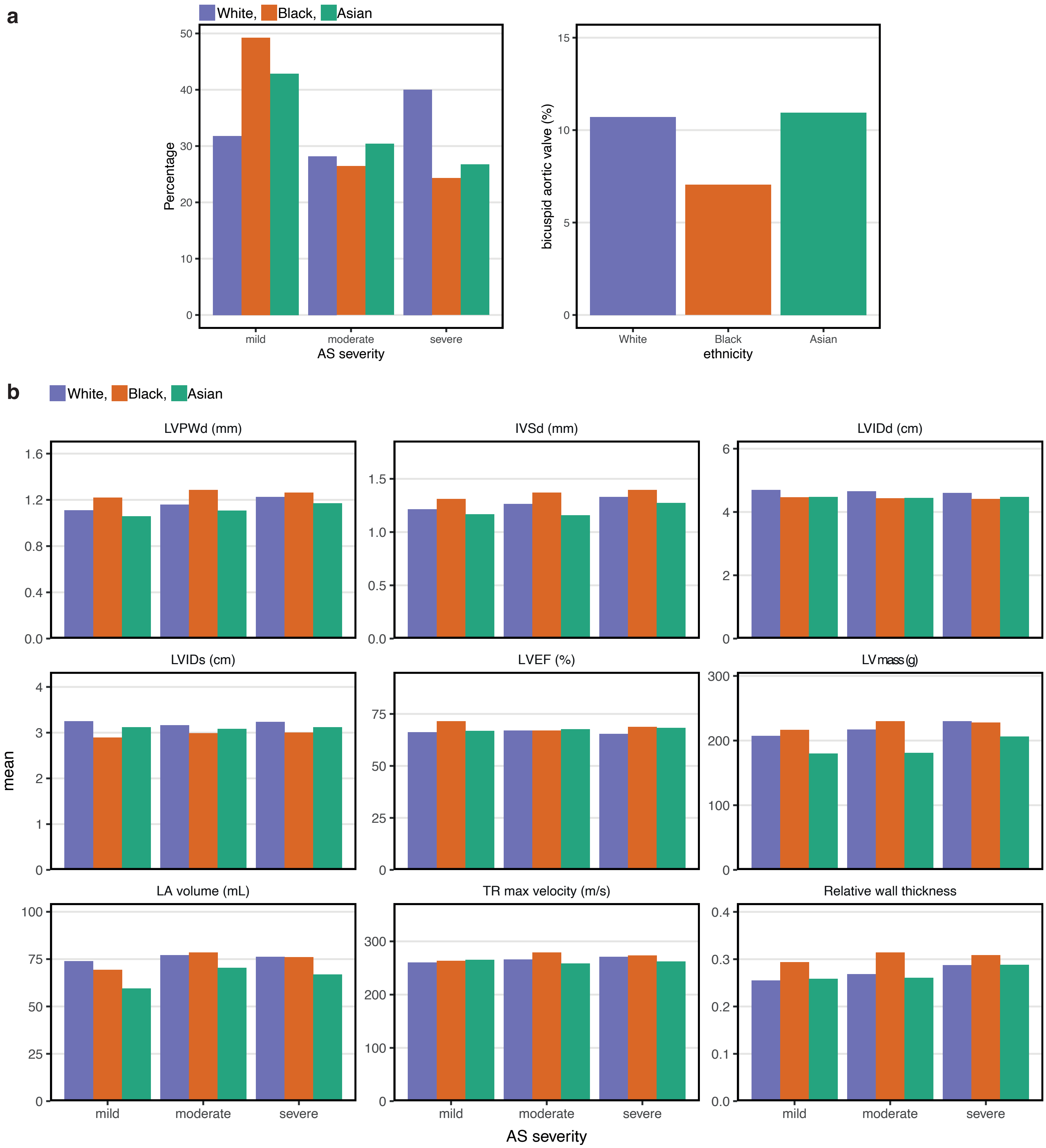

Supplementary Figure S3 | Echocardiogram variables stratified by ethnicity

a, Percentage bar-plots of severity at AS diagnosis (left), and bicuspid aortic valve (right) stratified by race and ethnicity.
b, Bar-plots showing mean echocardiogram parameters stratified by race and ethnicity and AS disease severity.
