# Supplementary material for: Racial and ethnic disparities in aortic stenosis within a universal healthcare system characterized by natural language processing for targeted intervention
Source: Eur Heart J Digit Health. 2025 Mar 18;6(3):392–403. doi: 10.1093/ehjdh/ztaf018 (PMC12088714; doi:10.1093/ehjdh/ztaf018)
Supplement: ztaf018_Supplementary_Data [file ztaf018_supplementary_data.zip › supplementary_4.pdf]

Supplementary Figure S4

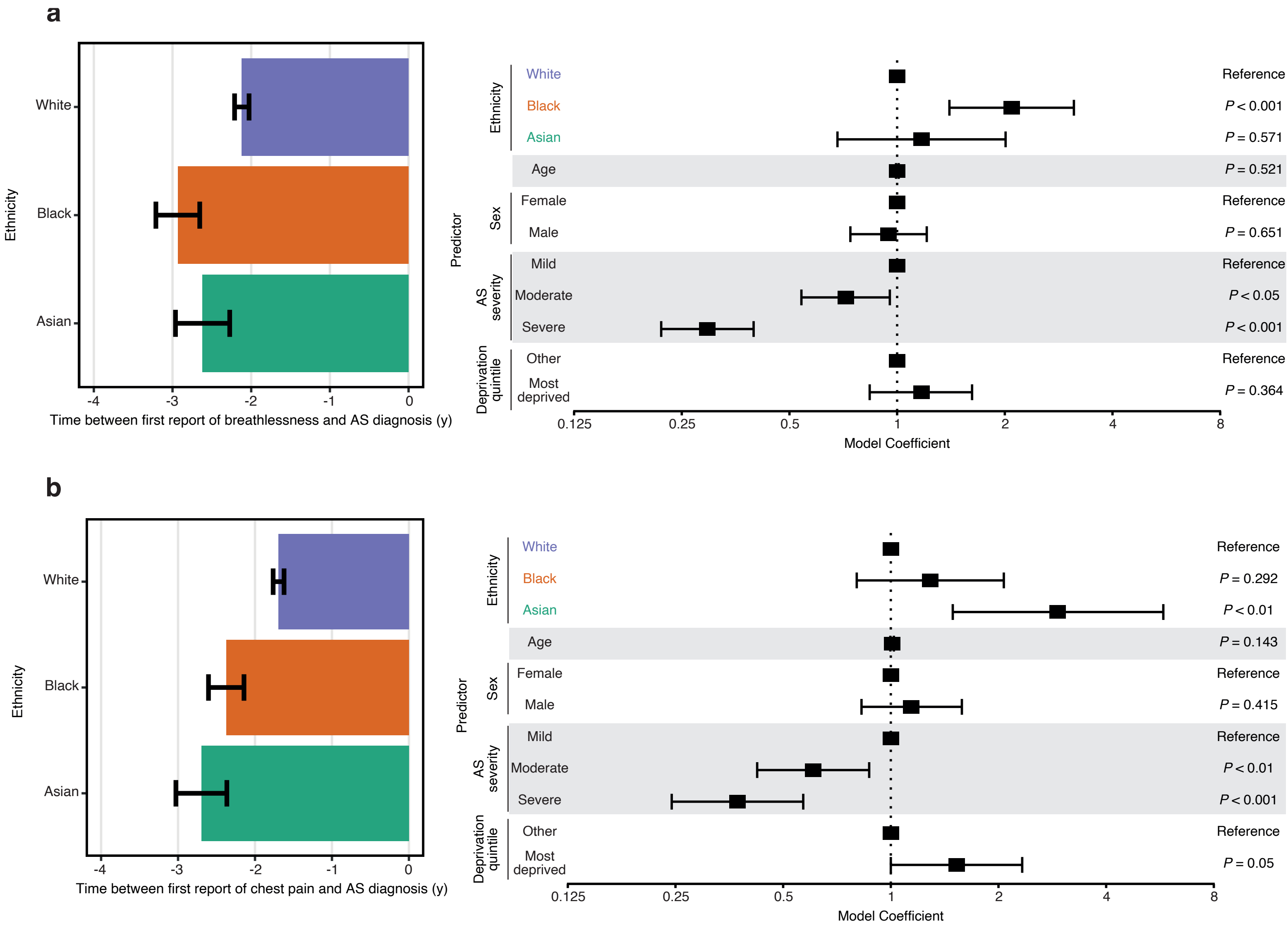

Supplementary Figure S4 | Time interval from symptoms to AS diagnosis

a, Time between first report of breathlessness symptoms and AS diagnosis.
b, Time between first report of chest pain symptoms and AS diagnosis.
Bar plots (left) shows mean time difference. Error bars for bar plots represent the standard error of the mean.
Forest plots (right) shows coefficients for a linear regression model adjusted for age, sex, AS disease severity and socioeconomic deprivation. Error bars for forest plots represent the limits of the 95% confidence interval for the model coefficient.
