# Supplementary material for: Racial and ethnic disparities in aortic stenosis within a universal healthcare system characterized by natural language processing for targeted intervention
Source: Eur Heart J Digit Health. 2025 Mar 18;6(3):392–403. doi: 10.1093/ehjdh/ztaf018 (PMC12088714; doi:10.1093/ehjdh/ztaf018)
Supplement: ztaf018_Supplementary_Data [file ztaf018_supplementary_data.zip › supplementary_5.pdf]

Supplementary Figure S5

a

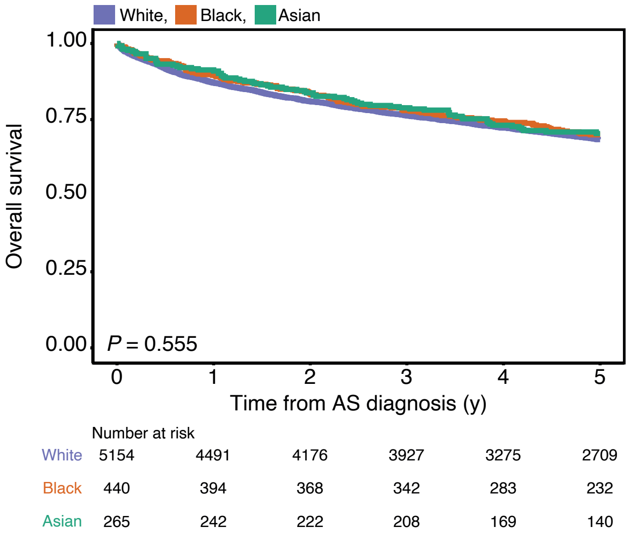

b

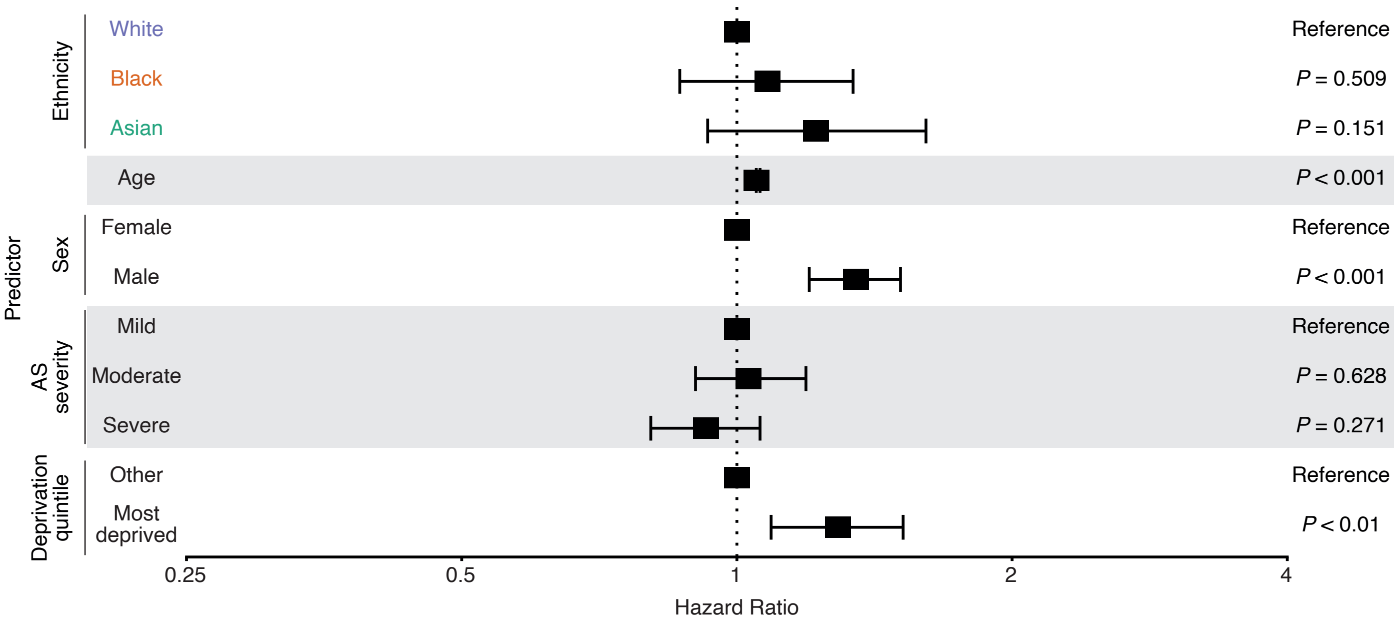

Supplementary Figure S5 | Mortality outcomes for all patients with AS

a, Kaplan-Meier plot showing overall survival outcomes from AS diagnosis stratified by ethnicity.
b, Forest plot showing adjusted hazard ratios for multivariate Cox analysis of overall survival outcomes from AS diagnosis stratified by ethnicity and adjusted for age, sex, socioeconomic deprivation and AS disease severity.
