# Supplementary material for: Racial and ethnic disparities in aortic stenosis within a universal healthcare system characterized by natural language processing for targeted intervention
Source: Eur Heart J Digit Health. 2025 Mar 18;6(3):392–403. doi: 10.1093/ehjdh/ztaf018 (PMC12088714; doi:10.1093/ehjdh/ztaf018)
Supplement: ztaf018_Supplementary_Data [file ztaf018_supplementary_data.zip › supplementary_6.pdf]

Supplementary Figure S6

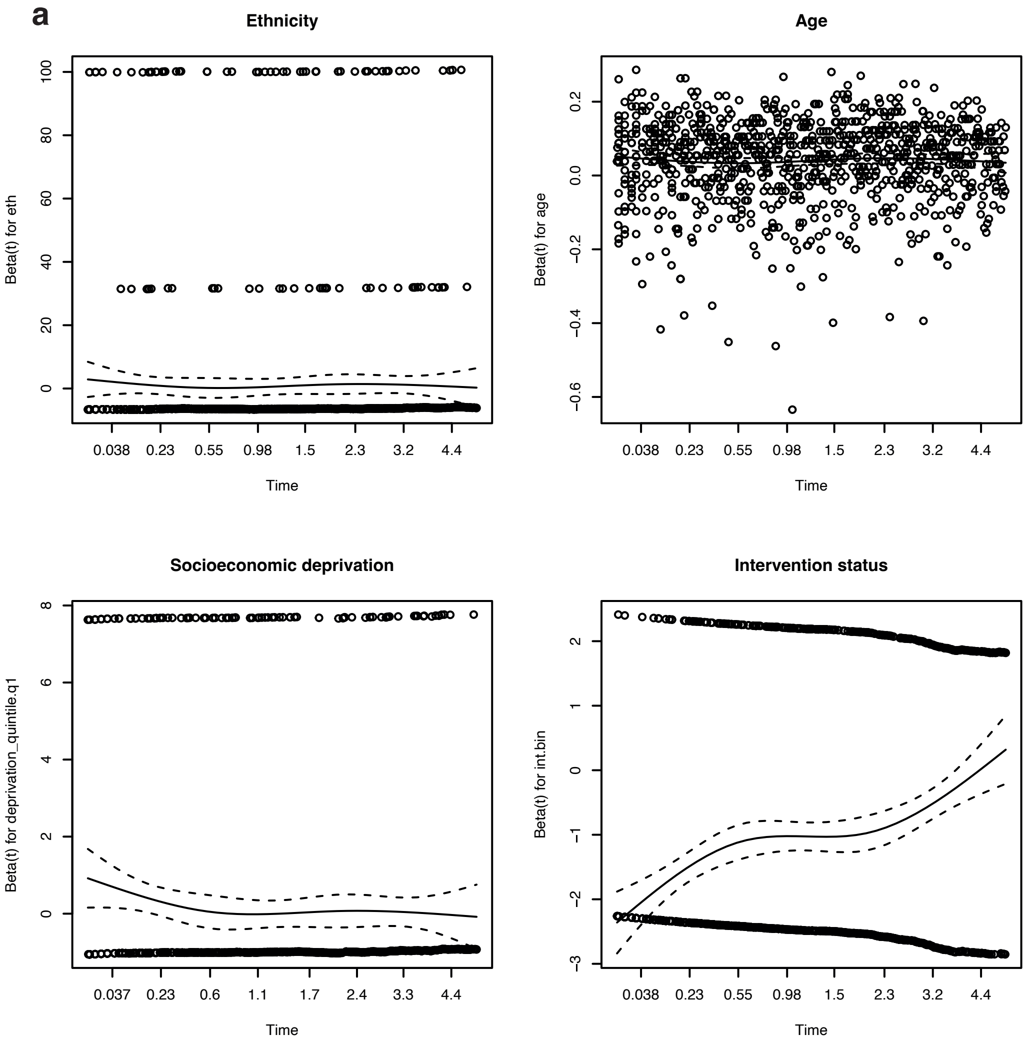

Supplementary Figure S6 | Mortality outcomes for patients with severe AS

a, Schoenfeld residuals to assess whether the Cox Proportional Hazards model assumptions were met.
